# Carcinoïd tumor revealing pernicious anemia

**DOI:** 10.11604/pamj.2014.19.153.4326

**Published:** 2014-10-15

**Authors:** Neirouz Ghannouchi Jaafoura, Ahlem Braham Krifa

**Affiliations:** 1 Department of Internal Medicine, Farhat Hached Hospital, Sousse, Tunisia; 2 Department of Gastro-Entérology, Sahloul Hospital, Sousse, Tunisia

**Keywords:** Carcinoïd tumor, pernicious anemia, atrophic gastritis

## Image in medicine

Endocrine tumors (ET) of the digestive tract are rare. Gastric carcinoid was the most common form of gastrointestinal ET's. It is a rare complication of pernicious anemia but may reveal the disease. We report the case of a 57-year- old women, wich is referred for exploration of epigastric pain with persistent dyspepsia. Gastroscopy found polypoids lesions (A) and gastric mucosae was enlarged, on histological study, by a well-circumscribed neoplastic proliferation covered by intact surface epithelium (B et C). Tumor cells have central, uniform nuclei, a low mitotic rate and co-express cytokeratine and chromogranine A, concluding to well-differentiated neuroendocrine tumor. Pallor on examination led us to perform NFS showing aregenrative anemia (hemoglobin at 7.2 g/dl) with high mean corpuscular volume and megaloblastosis in myelogram. Serum vitamine B 12 level is 60 pg /l and homocysteine level is 57 μmol/l. Vitamin replacement treatment is given as well as endoscopic resection of polyps since assessment of extension is negative. The onset of pernicious anemia is difficult to establish because of its insidious presentation. This slow progression of vitamine B 12 deficiency, resulting from advanced autoimmune atrophic gastritis, explains the adaptation to anemic syndrome that does not represent the main complaint in this patient. This case confirm the importance of endoscopic evaluation at the diagnosis of pernicious anemia.

**Figure 1:**
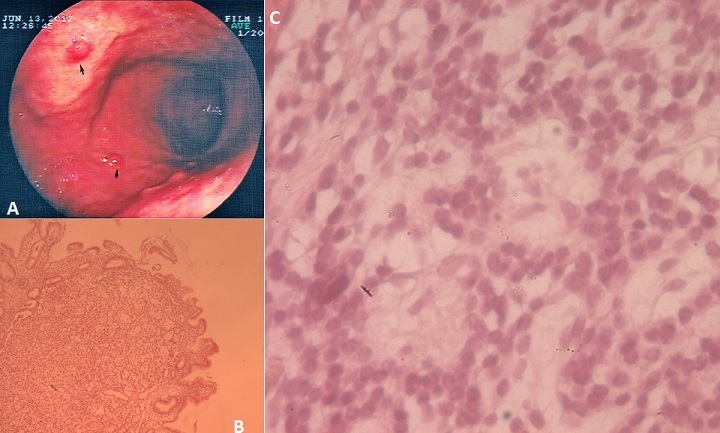
(A) polypoid lesions on stomach; (B) neoplastic proliferation on gastric mucosae; (C) tumor cells have central, uniform nuclei and a low mitotic rate

